# Supplementary material for: The local mechanostructural properties of protein cargoes regulate nucleocytoplasmic transport
Source: Nat Phys. 2026 Apr 30;22(5):770–83. doi: 10.1038/s41567-026-03242-2 (PMC13183583; doi:10.1038/s41567-026-03242-2)
Supplement: Supplementary file 1 — Supplementary Figs. 1–28, Table 1 and references. [file 41567_2026_3242_MOESM1_ESM.pdf]

nature physics

Supplementary information https://doi.org/10.1038/s41567-026-03242-2

The local mechanostructural properties of protein cargoes regulate nucleocytoplasmic transport

In the format provided by the authors and unedited

Table of Contents:

-Supplementary Figures S1-S28
-Supplementary Table S1
-Supplementary References.

Supplementary Figures:

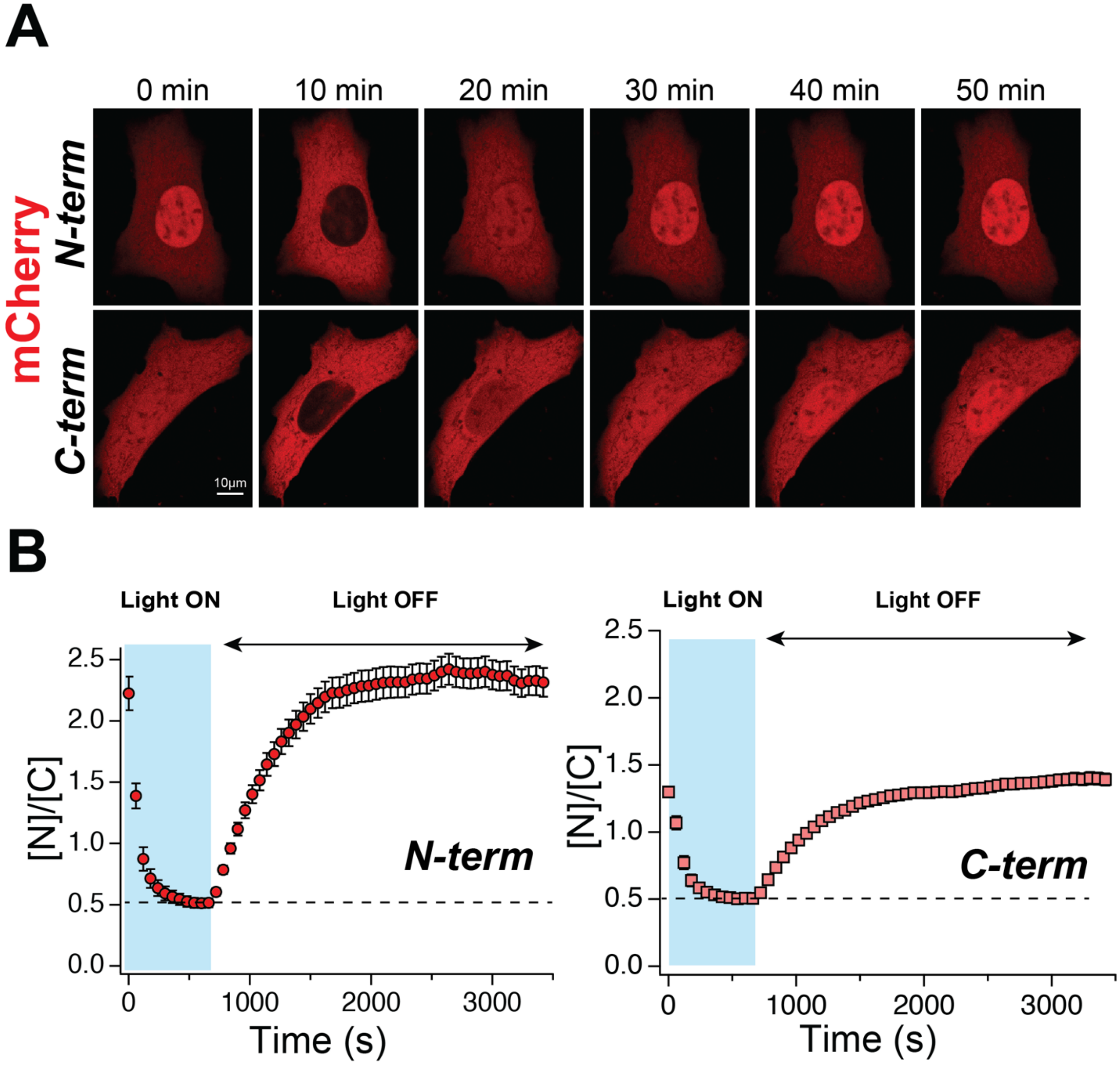

Figure S1. Time courses of the optogenetics experiment for the "Import forward" mCherry and "Import reverse" mCherry constructs. (A) Confocal images of representative U2OS cells overexpressing the "Import forward" mCherry (upper) and "Import reverse" mCherry (lower) optogenetic constructs at different time frames. Scale bar 10 µm. (**B**) Average nucleus-to-cytoplasm localisation, including the stimulation phase, for the "Import forward" mCherry (left) and "Import reverse" mCherry (right). Upon blue light exposure, the relative nucleus-to-cytoplasm signal decreases to comparable levels, indicating that the performance of the optogenetic construct is independent of the protein orientation. *n*=86 (*N-term*); *n*=69 (C-term) from *N>3* independent experiments.

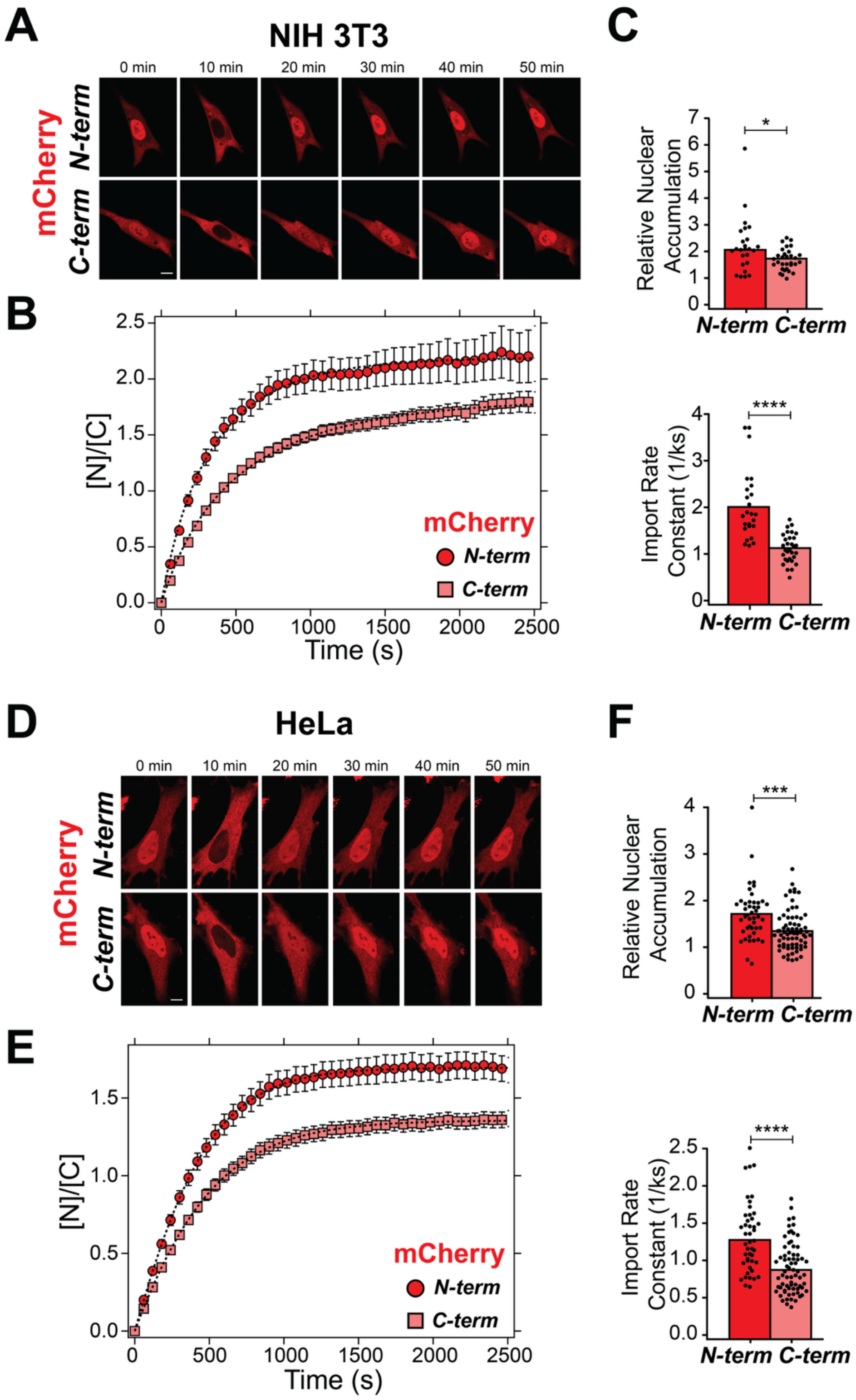

Figure S2. The asymmetric nuclear import kinetics of mCherry are maintained in different cell lines. (A-C) Nuclear import kinetics of mCherry when translocating using the "Import forward" or "Import reverse" constructs measured in NIH 3T3 cells. **(A)** Representative confocal images of NIH 3T3 cells during the export and the recovery phase, scale

bar 10 µm. **(B)** Average time-courses of the nucleus-to-cytoplasm localisation of the “Import forward” and “Import reverse” mCherry. (**C**) Relative nuclear accumulation (upper) and import rate (lower) calculated from fits to the recovery time courses. Bars indicate mean±SEM. Accumulation: N-term K_e=2.21±0.20; C-term K_e=1.69±0.07 Import rate: N-term k_I=2.04±0.15 ks^{-1}; C-term k_I=1.12±0.06. ks^{-1}. Significance levels for two-tailed Mann-Whitney non-parametric test. Accumulation, *P*=0.04; Import rate, *P*=$5.2x10^{-7}$. *n*=26 (N-term); *n*=32. (C-term). (**D-F**) Nuclear import kinetics of mCherry for the “Import forward” or “Import reverse” constructs, measured in HeLa cells. **(D)** Representative confocal images of HeLa cells during the export and the recovery phase, scale bar 10 µm. **(E)** Average time-courses of the nucleus-to-cytoplasm localisation of the import “Import forward” and “Import reverse” mCherry. (**F**) Relative nuclear accumulation (upper) and import rate (lower) calculated from fits to the recovery time courses. Bars indicate mean±SEM. Accumulation: N-term K_e=1.71±0.08; C-term K_e=1.37±0.05. Import rate: N-term k_I= 1.31±0.07 ks^{-1}; C-term k_I=0.90±0.04 ks^{-1}. Significance levels for two-tailed Mann-Whitney non-parametric test. Accumulation, *P*=$2.0x10^{-4}$; Import rate, *P*=$2.0x10^{-6}$. *n*=45 (N-term); *n*=71 (C-term).

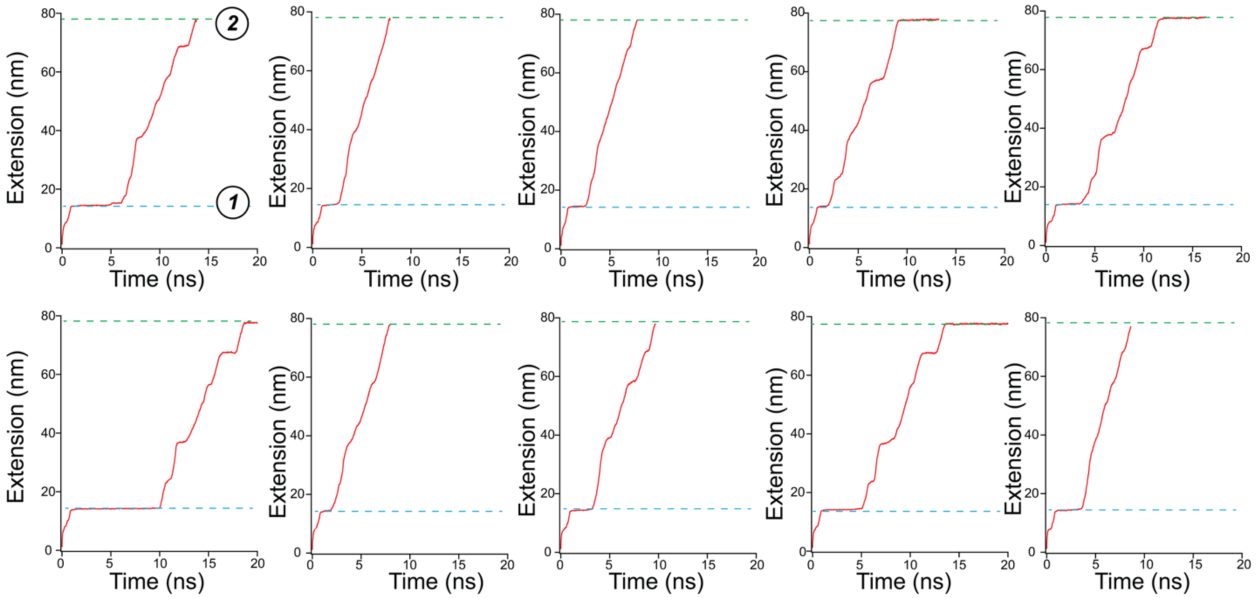

Figure S3. Representative SMD trajectories of mCherry under a constant force of 350 pN. In all 10 simulations, the unfolding pathway of mCherry shows an intermediate with an extension of ∼18 nm (blue line), which corresponds to the unfolding of its N-term region, to then unravel the remainder of the structure to reach the fully stretched state (green line).

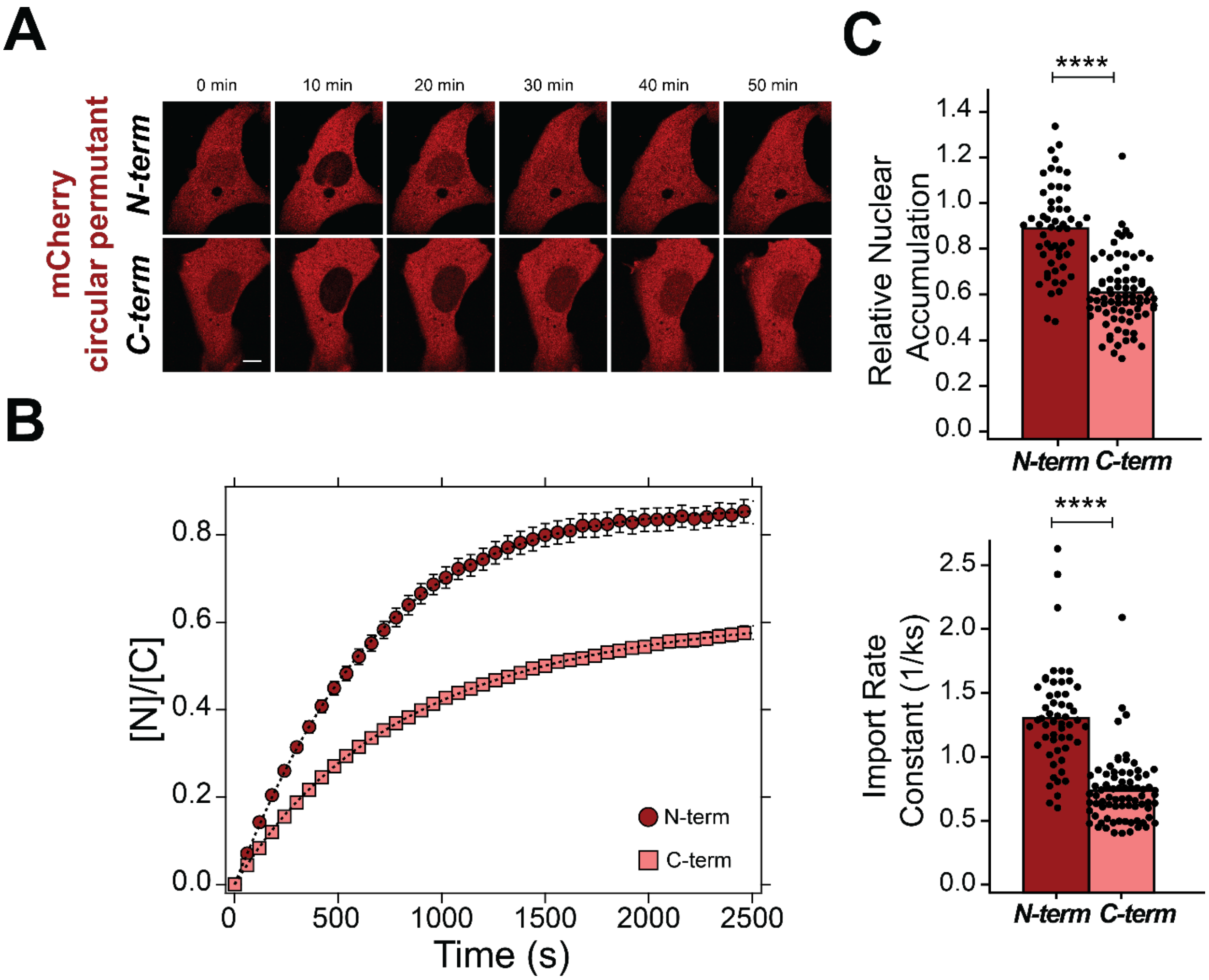

Figure S4. Asymmetric nuclear import in mCherry circular permutant. (A) Confocal images of representative U2OS cells across an optogenetics experiment for the "Import forward" (upper) and "Import reverse" (lower) mCherry circular permutant, scale bar 10 µm. **(B)** Average time-courses of the nucleus-to-cytoplasm localisation of the "Import forward" and "reverse" mCherry circular permutant. **(C)** Relative nuclear accumulation (upper) and import rate (lower) calculated from fits to the recovery time courses. Bars indicate mean±SEM. Accumulation: N-term K_e=0.89±0.03; C-term K_e=0.60±0.02; Import rate: N-term k_I=1.30±0.05 ks^{-1}; C-term k_I=0.73±0.03. ks^{-1}. Significance levels for two-tailed Mann-Whitney non-parametric test. Accumulation, *P*=9.0x10^{-14}; Import rate, *P*=6.7x10^{-16}. *n*=54 (N-term); *n*=77 (C-term) from *N>3* independent experiments.

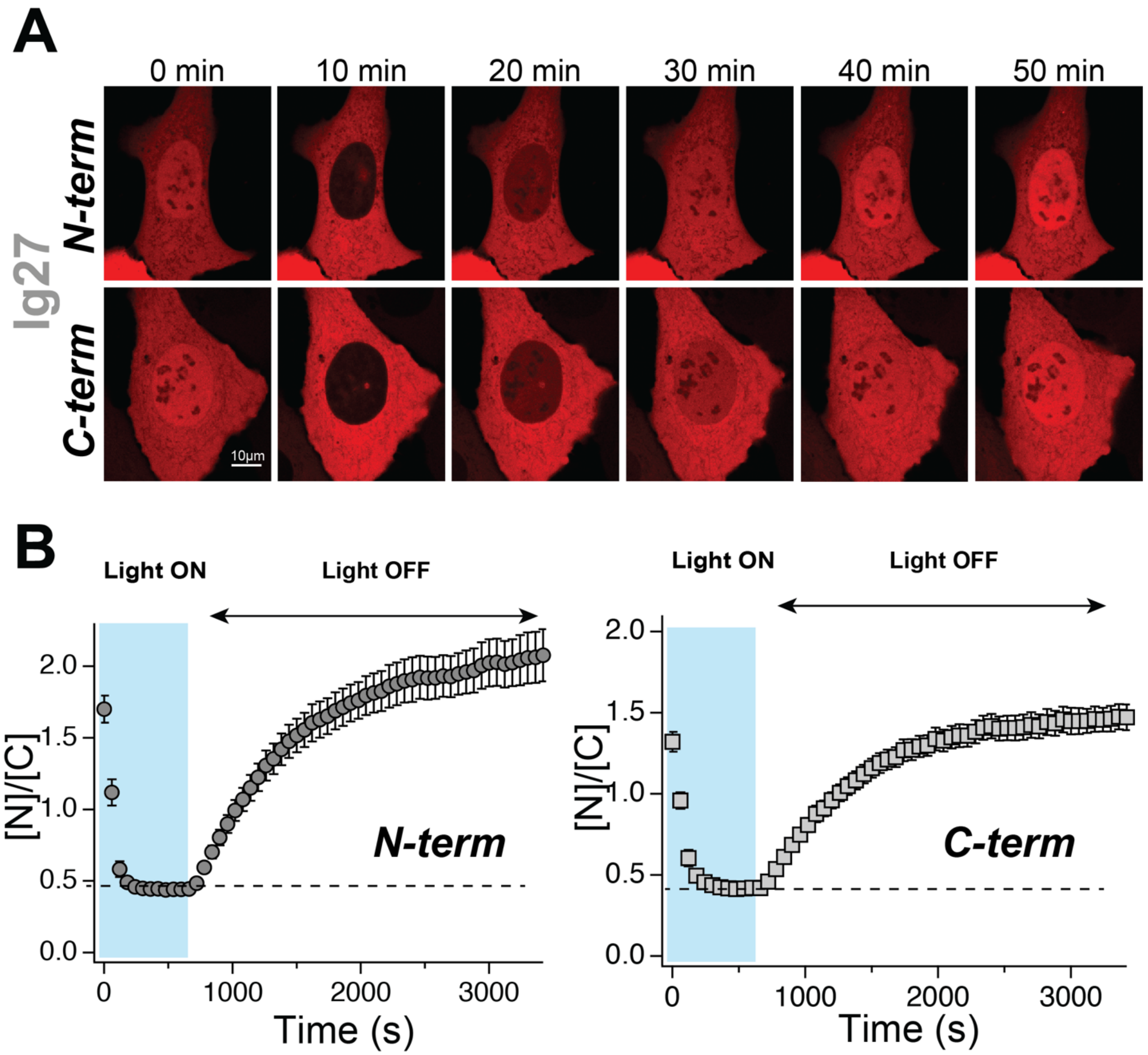

Figure S5. Time courses of the optogenetics experiment for the "Import forward" Ig27 and "Import reverse" Ig27 constructs. (A) Confocal images of representative U2OS cells overexpressing the "Import forward" Ig27 (upper) and "Import reverse" Ig27 (lower) optogenetic constructs at different time frames. Scale bar 10 µm. (**B**) Average nucleus-to-cytoplasm localisation, including the stimulation phase, for the "Import forward" Ig27 (left) and "Import reverse" Ig27 (right) optogenetic constructs. *n*=64 (N-term); *n*=64 (C-term) from *N>3* independent experiments.

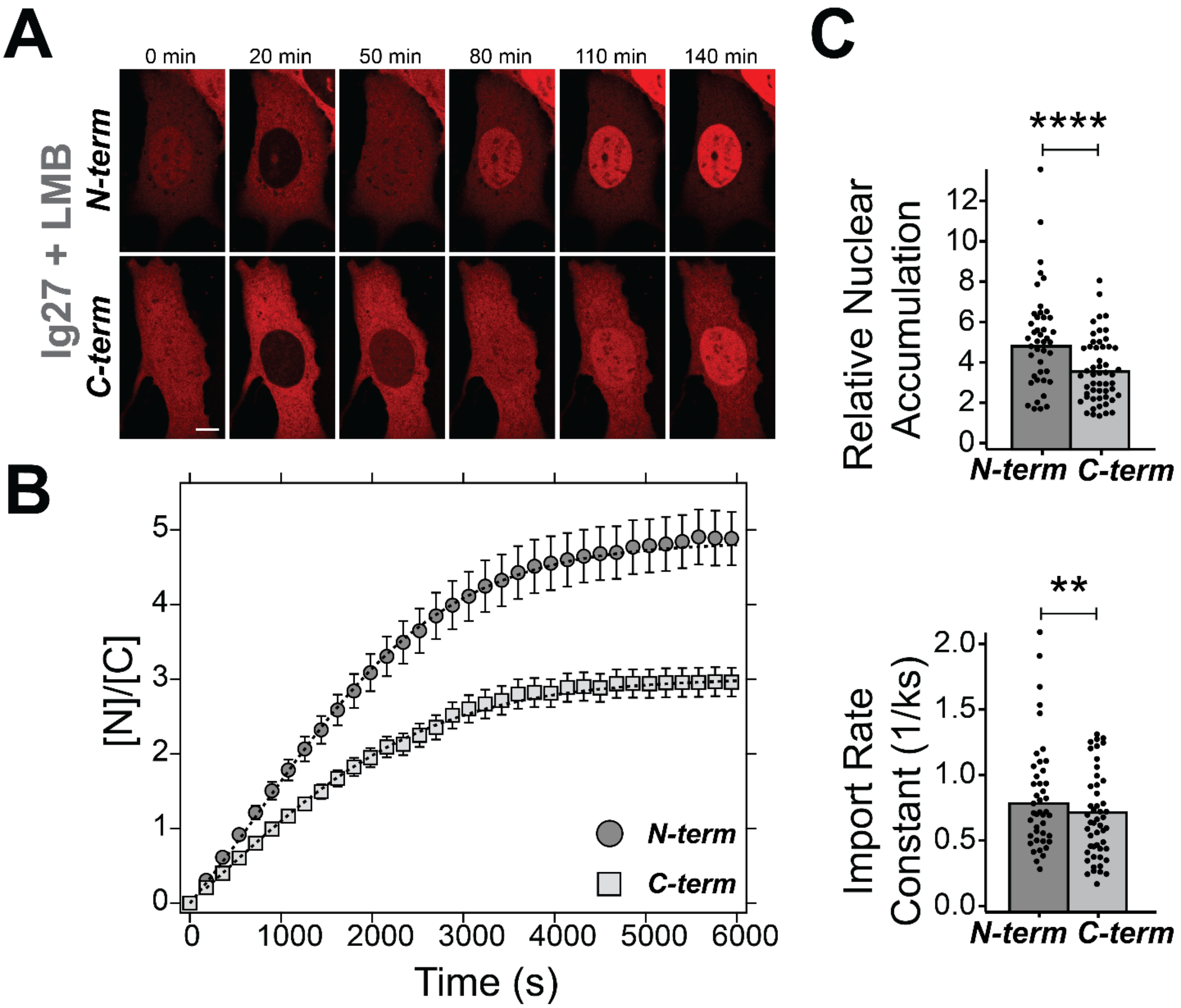

Figure S6. Blocking active nuclear export with leptomyocin maintains the asymmetric nuclear import kinetics of Ig27. (**A-C**) Nuclear import kinetics of Ig27 when translocating from the N- or C-terminus in the presence of 20 nM leptomyocinB. **(A)** Representative confocal images of U2OS cells during the export and the recovery phase, scale bar 10 µm. **(B)** Average time-courses of the nucleus-to-cytoplasm localisation of the "Import forward" and "Import reverse" Ig27. (**C**) Relative nuclear accumulation (upper) and import rate (lower) calculated from fits to the recovery time courses. Bars indicate mean±SEM. Accumulation: N-term K_e=5.19±0.38; C-term K_e=3.62±0.23. Import rate: N-term k_I=0.85±0.06 ks^{-1}; C-term k_I=0.68±0.05 ks^{-1}. Significance levels for two-tailed Mann-Whitney non-parametric test. Accumulation, P=7.7x10^{-5}; Import rate, P=4.6x10^{-3}. Scale bar 10 µm. n=40 (N-term); n=47 (C-term) from N>3 independent experiments.

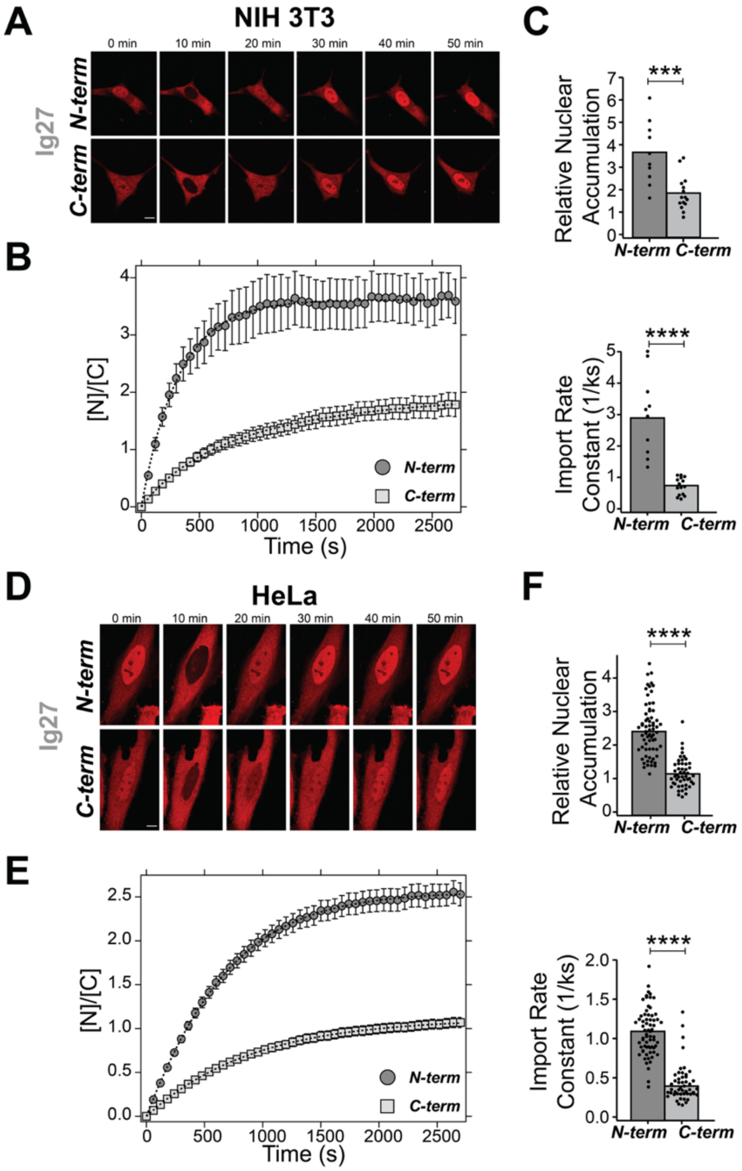

Figure S7. The asymmetric nuclear import kinetics of Ig27 are maintained in different cell lines. (A-C) Nuclear import kinetics of Ig27 when translocating from the N- or C-terminus measured in NIH 3T3 cells. **(A)** Representative confocal images of NIH 3T3 cells during the export and the recovery phase, scale bar 10 µm. **(B)** Average time-courses of the nucleus-to-cytoplasm localisation of the "Import forward" and "Import reverse" Ig27. (**C**) Relative nuclear accumulation (upper) and import rate (lower) calculated from fits to the recovery time courses. Bars indicate mean±SEM. Accumulation: N-term K_e=3.63±0.44; C-term K_e=1.82±0.20 Import rate: N-term k_I=2.98±0.41 ks^{-1}; C-term k_I=0.71±0.07. ks^{-1}. Significance levels for two-tailed Mann-Whitney non-parametric test. Accumulation, P=9.0x10^{-4}; Import rate, P=6.1x10^{-7}. n=10 (N-term); n=15 (C-term). (**D-F**) Nuclear import kinetics of Ig27 when translocating from the N- or C-terminus measured in HeLa cells. **(D)** Representative confocal images of HeLa cells during the export and the recovery phase, scale bar 10 µm. **(E)** Average time-courses of the nucleus-to-cytoplasm localisation of the "Import forward" and "Import reverse" Ig27. (**F**) Relative nuclear accumulation (upper) and import rate (lower) calculated from fits to the recovery time courses. Bars indicate mean±SEM. Accumulation: N-term K_e=2.56±0.13; C-term K_e=1.15±0.05. Import rate: N-term k_I= 1.11±0.04 ks^{-1}; C-term k_I=0.42±0.03 ks^{-1}. Significance levels for two-tailed Mann-Whitney non-parametric test. Accumulation, P=0; Import rate, P=1.3x10^{-15}. n=68 (N-term); n=49 (C-term).

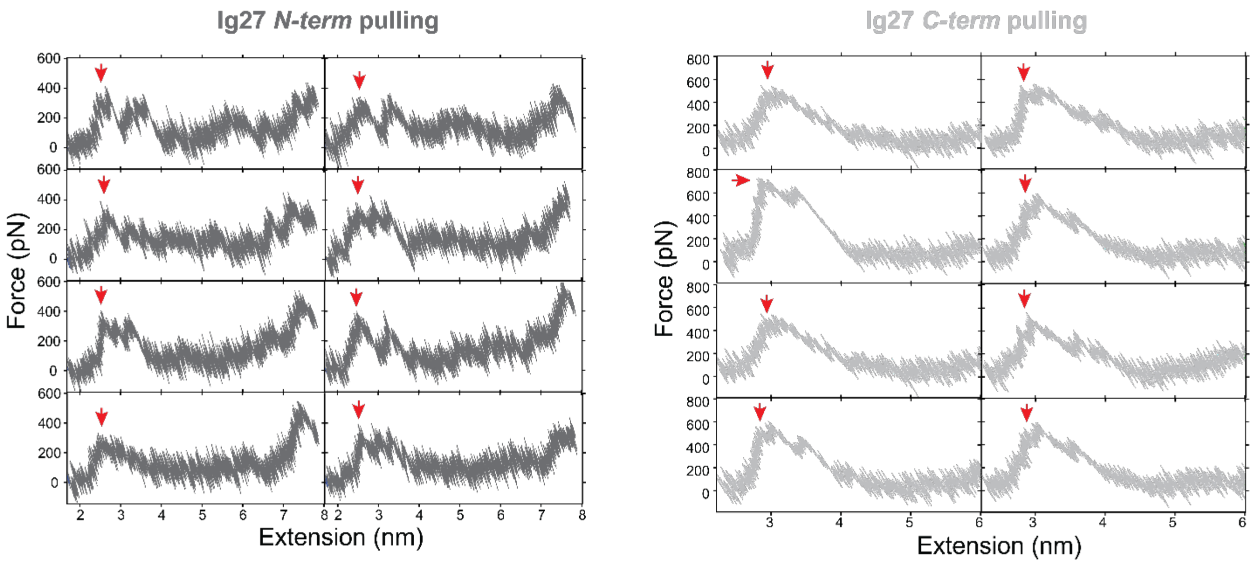

Figure S8. Representative unfolding trajectories of Ig27 pulled from the N-term (left) and C-term (right). In each case, the rupture peak corresponded to the unravelling of the N-terminal or C-terminal strand, respectively, and has been marked with an arrow.

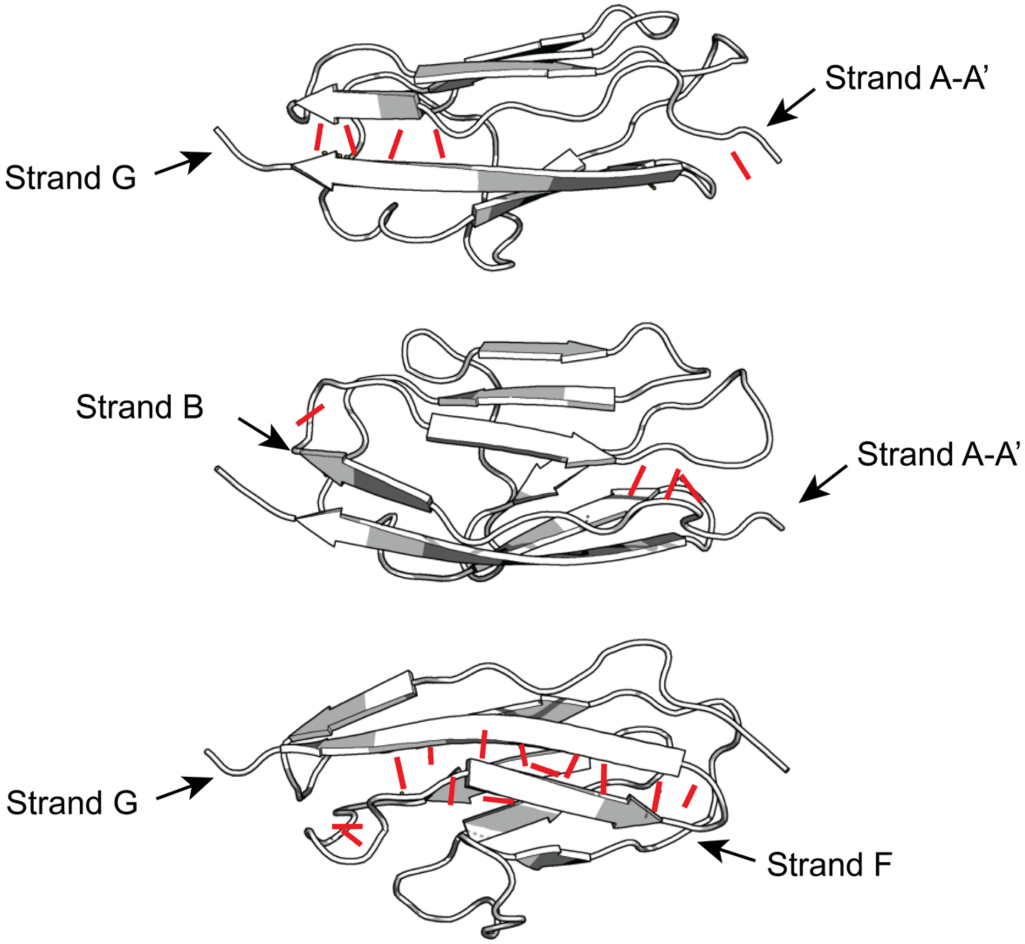

Figure S9. Cartoon representation of the Ig27 highlighting the H-bonds formed between its β-strands. (upper) The mechanical clamp formed between strands A-A’ and G shows 4 hydrogen bonds; (middle) The N-term region characterised by the hairpin formed between strands A-A’ and strand B shows 3 hydrogen bonds; (lower) the C-terminal hairpin formed between strand F and G shows 8 hydrogen bonds. Peeling the C-term strand results in the rupture of a higher number of hydrogen bonds than the N-terminal strand. Hydrogen bonds identified in Pymol using a cutoff distance of 4 Å.

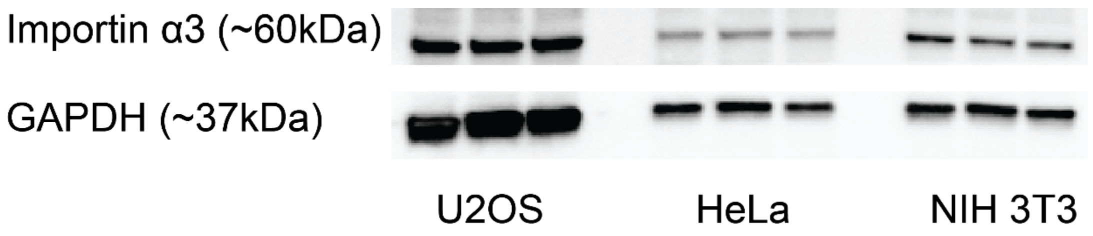

Figure S10. Expression of Importin α3 in U2OS, HeLa, and NIH 3T3 cells. Representative immunoblotting of importin α3 relative to GAPDH expression.

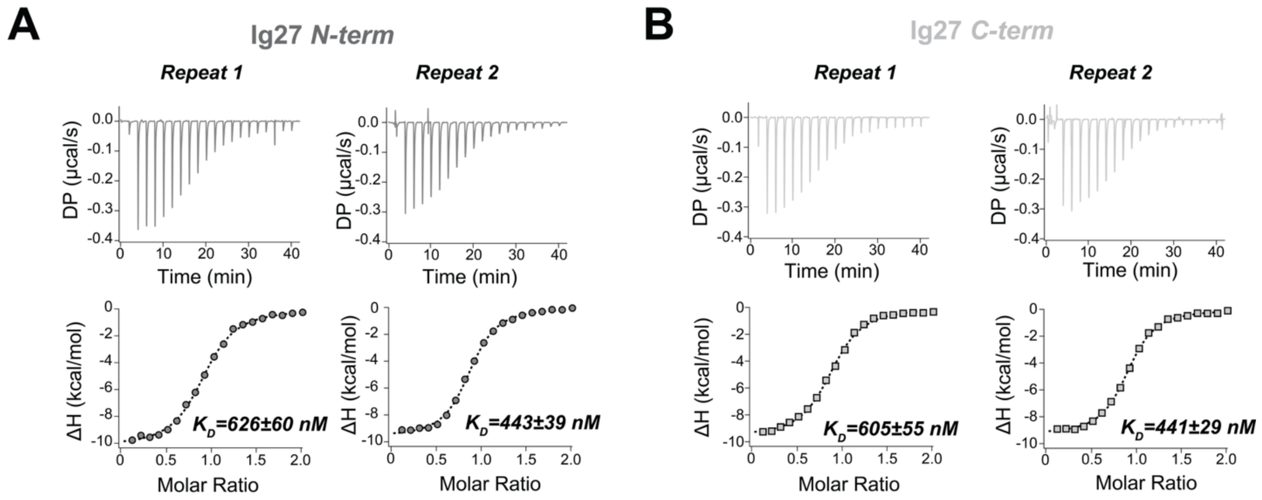

Figure S11. Titration experiments of NLS-Ig27 (A) and Ig27-NLS (B) into importin α3. Top panels show the thermogram for injection, and bottom panels show the incremental enthalpy changes, corrected for heats of dilution, fitted to a one-site binding model. Ig27 is injected at 17 µM into 170 µM of importin α3. Average affinity constants: K_D=535±129 nM (N-term binding) and K_D=523±116 nM (C-term binding). Thermodynamic parameters from data analysis are shown in Table S1.

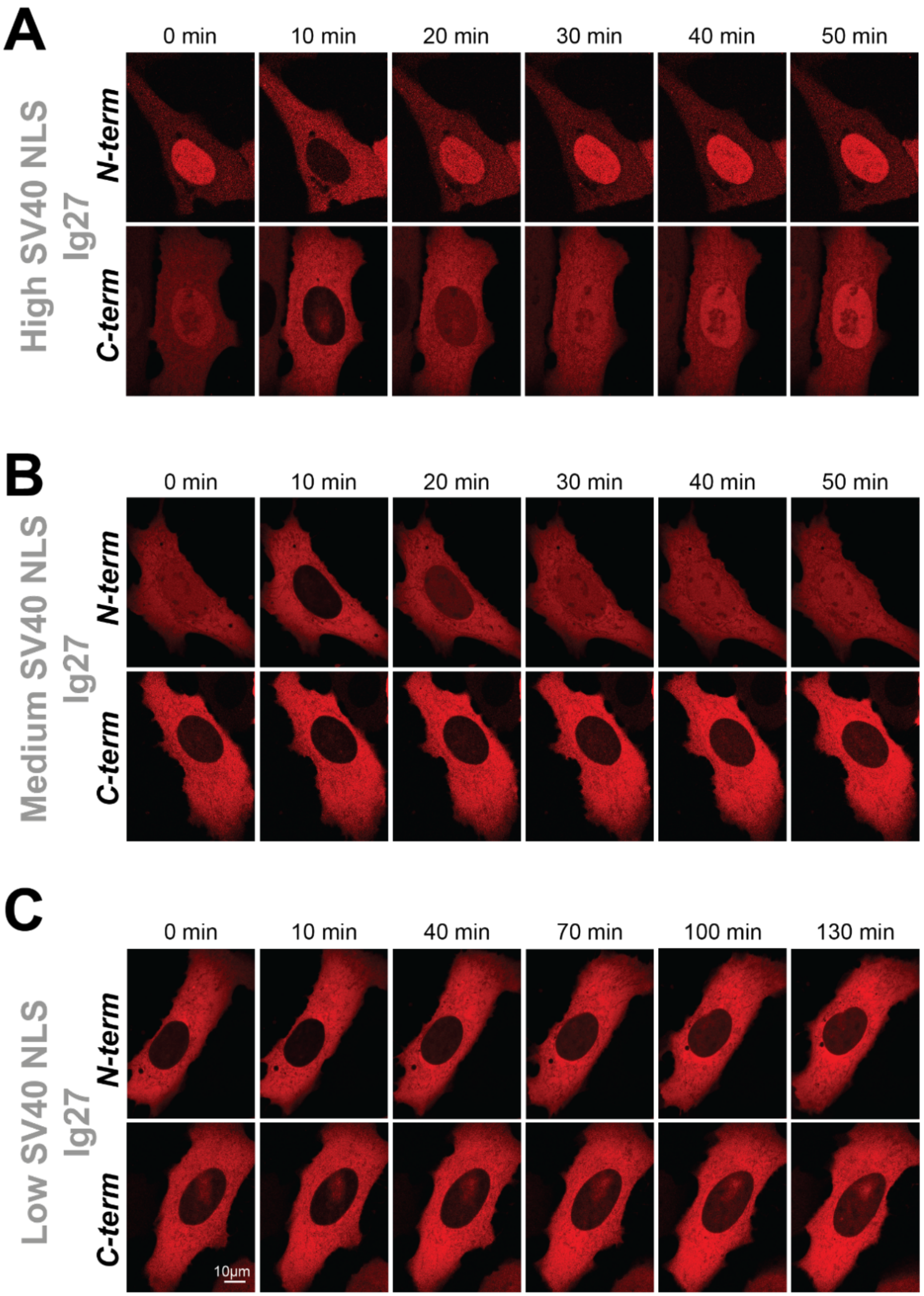

Figure S12. Confocal images of representative U2OS cells overexpressing the N-term Ig27 (upper) and C-term Ig27 (lower) "Import forward" and "Import reverse" constructs with the high (A), medium (B), and low (C) affinity SV40 NLS at different time frames. Scale bar 10 μm.

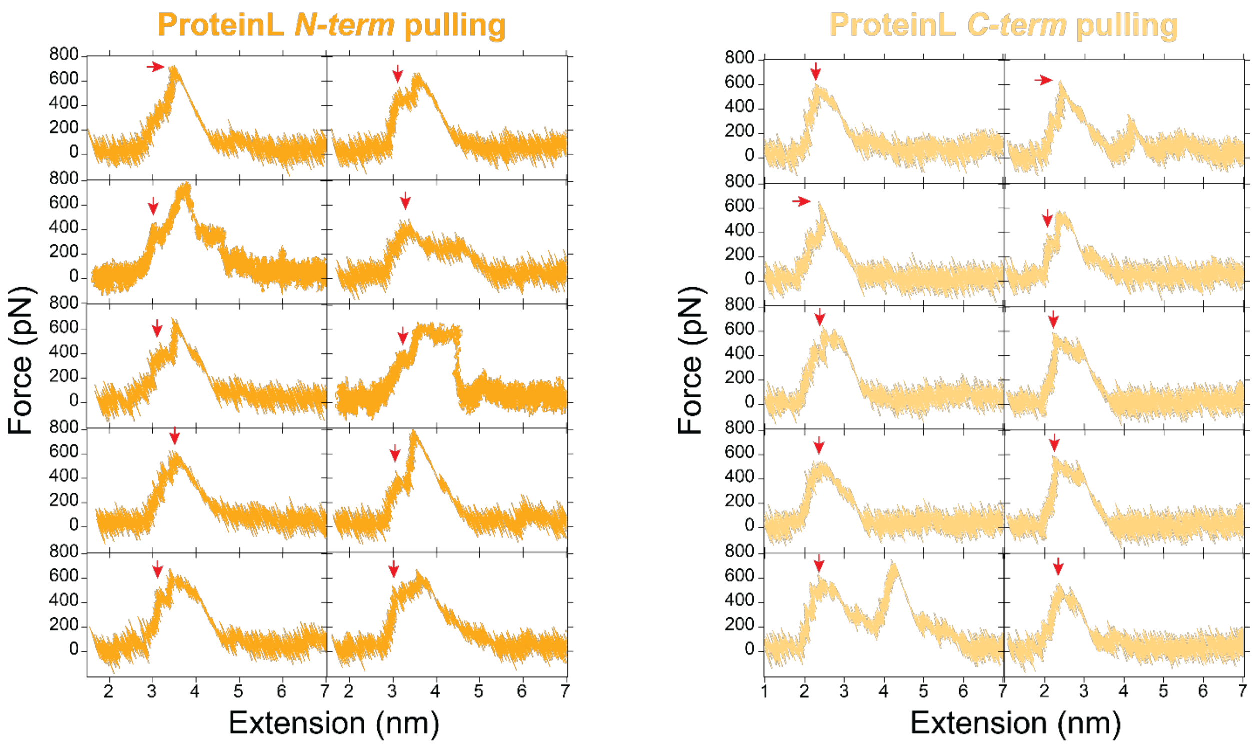

Figure S13. Molecular Dynamics unfolding trajectories of Protein L pulled from the N-term (left) and C-term (right). In each case, the first rupture peak corresponded to the unravelling of the N-terminal or C-terminal strand, respectively. The main measured peak has been indicated with an arrow.

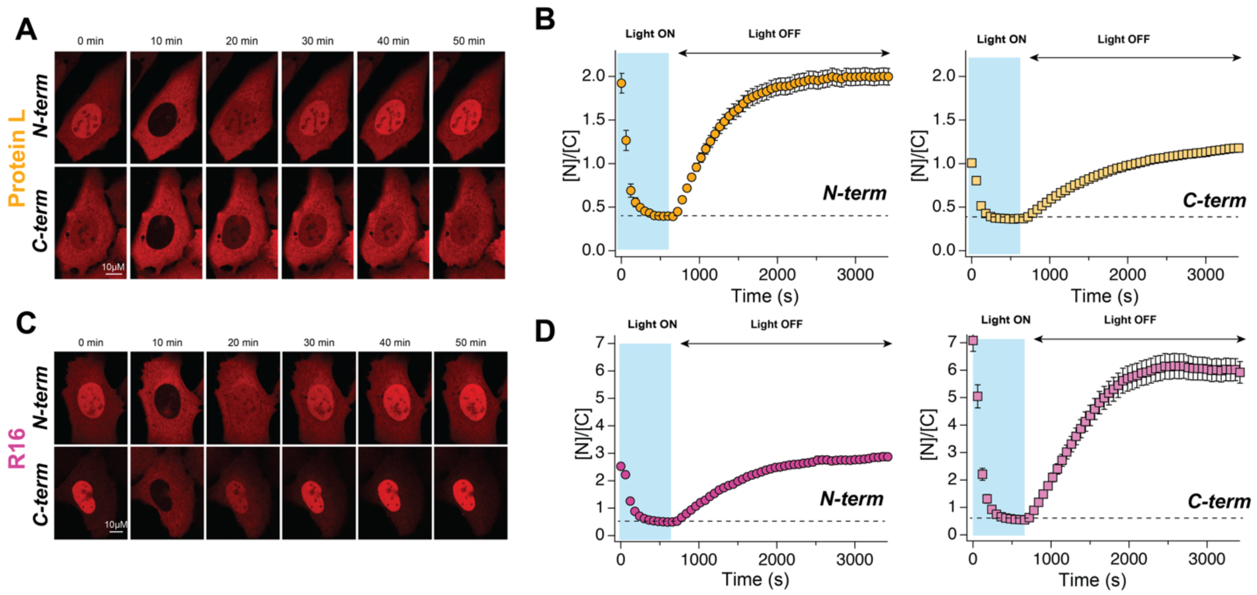

Figure S14. Time courses of the optogenetics experiment for the "Import forward" protein L and "Import reverse" protein L constructs (A-B) and "Import forward" R16 and "Import reverse" R16 (C-D). (A) Confocal images of representative U2OS cells overexpressing the "Import forward" Protein L (upper) and "Import reverse" Protein L (lower) optogenetic constructs at different time frames. Scale bar 10 µm. (**B**) Average nucleus-to-cytoplasm localisation, including the stimulation phase, for the "Import forward" Protein L (left) and "Import reverse" Protein L (right). *n*=129 (N-term); *n*=116 (C-term) from *N>3* independent experiments. **(C)** Representative confocal images for "Import forward" R16 (upper) and "Import reverse" R16 (lower). **(D)** Average nucleus-to-cytoplasm localisation for the "Import forward" R16 (left) and "Import reverse" R16 (right). *n*=110 (N-term); *n*=119 (C-term) from *N>3* independent experiments.

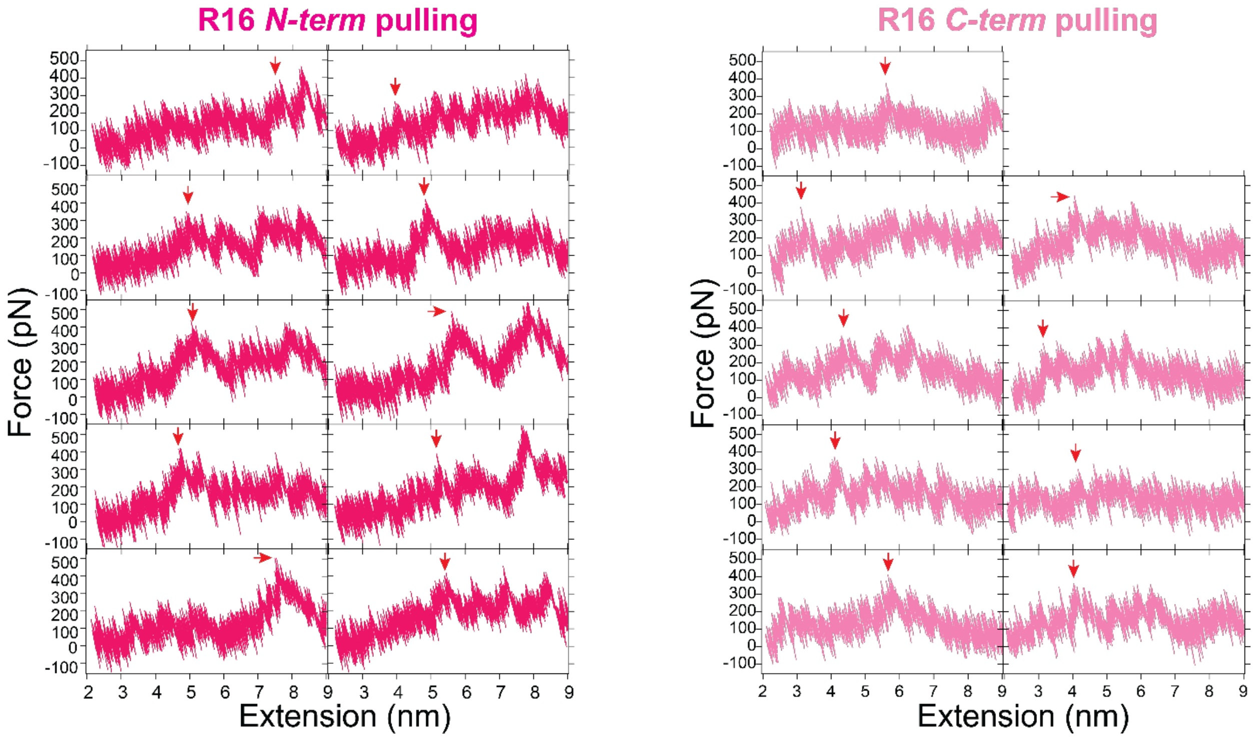

Figure S15. Molecular Dynamics unfolding trajectories of R16 pulled from the N-term (left) and C-term (right). In each case, the first rupture peak corresponded to the unravelling of the N-terminal or C-terminal strand, respectively. The first rupture peak has been marked with an arrow.

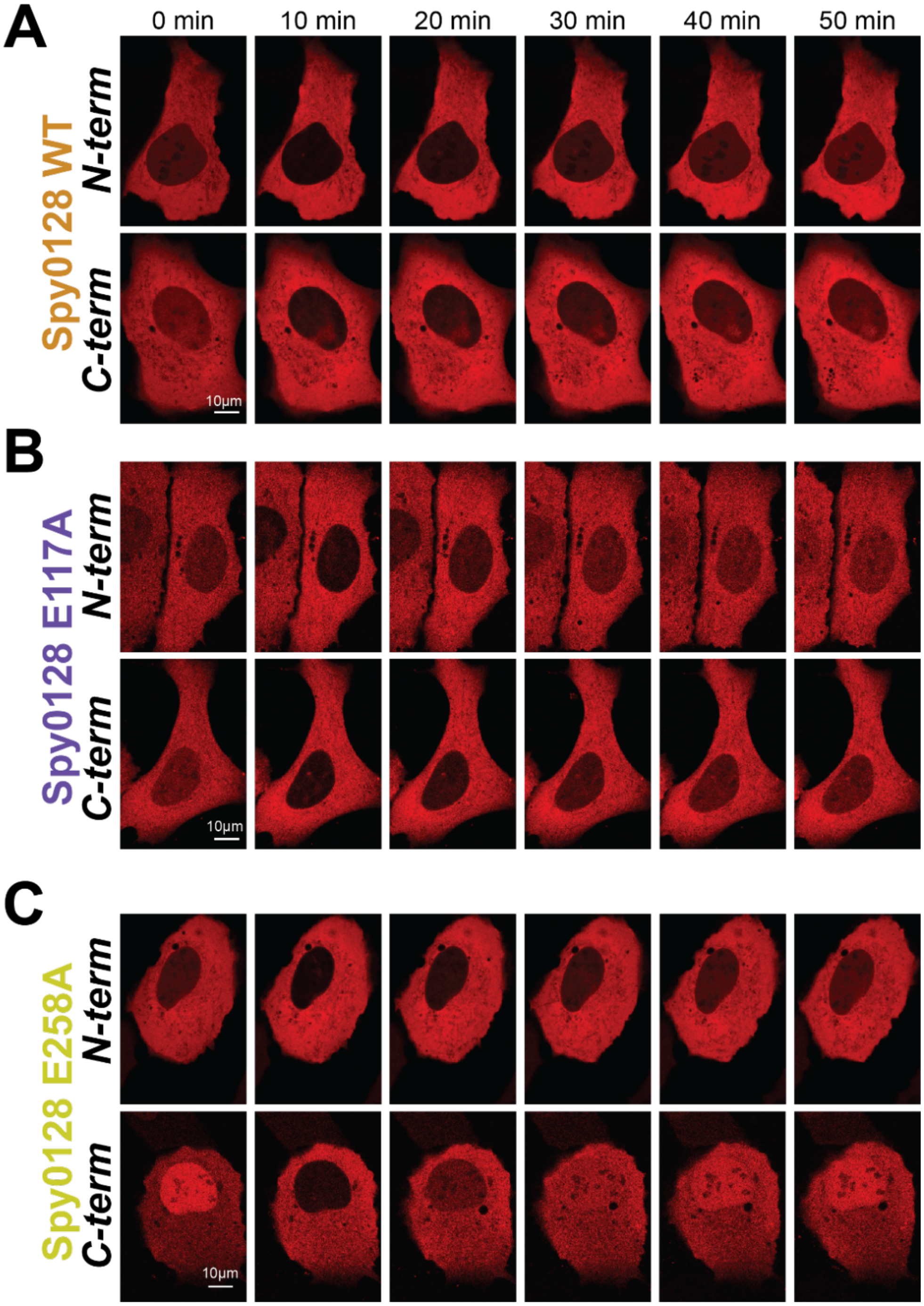

Figure S16. Confocal images of representative U2OS cells overexpressing the "Import forward" (upper) and "Import reverse" constructs (lower) of Spy0128WT (A), Spy0128 E117A (B), and Spy0128 E258A (C) at different time frames.

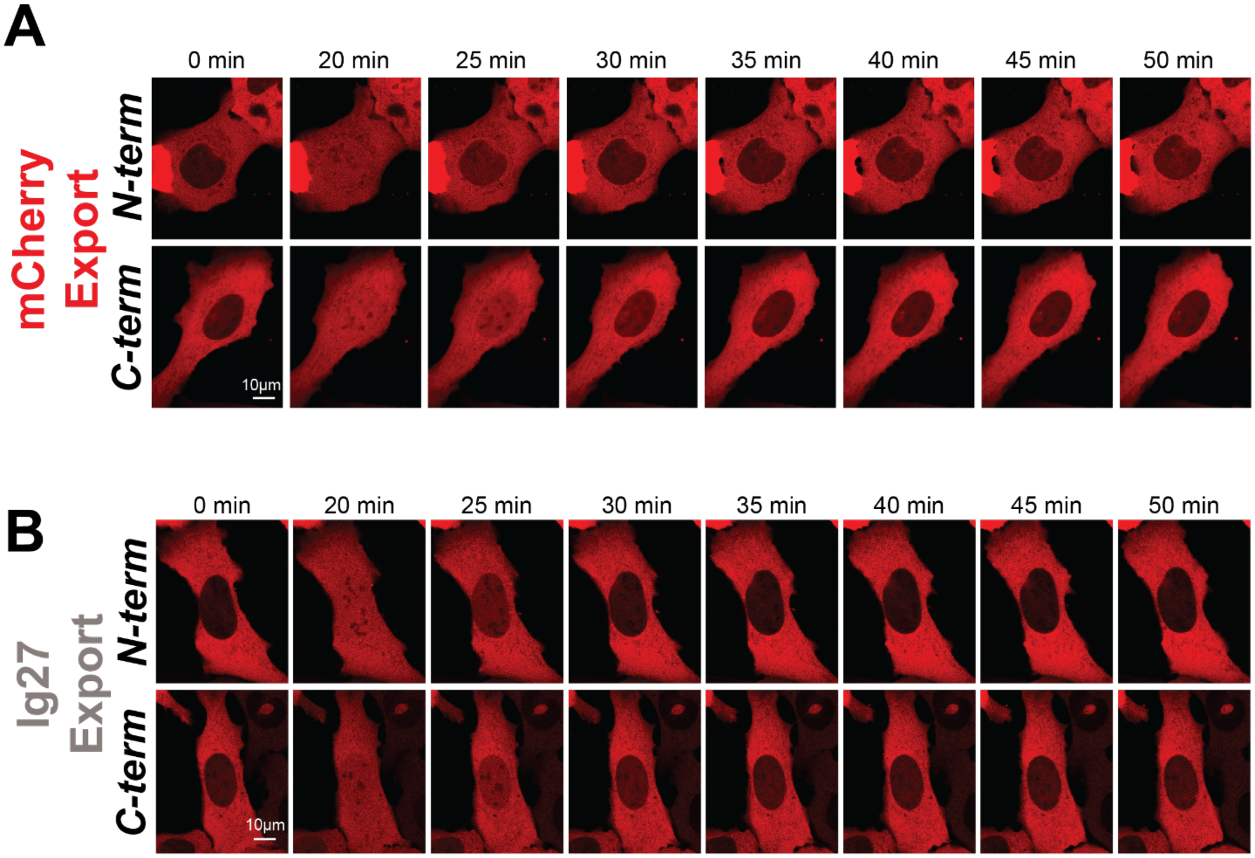

Figure S17. Confocal images of representative U2OS cells overexpressing the (A) N-term (upper) and C-term (lower) mCherry and (B) N-term (upper) and C-term (lower) Ig27 "Export forward" and "Export reverse" constructs at different time frames.

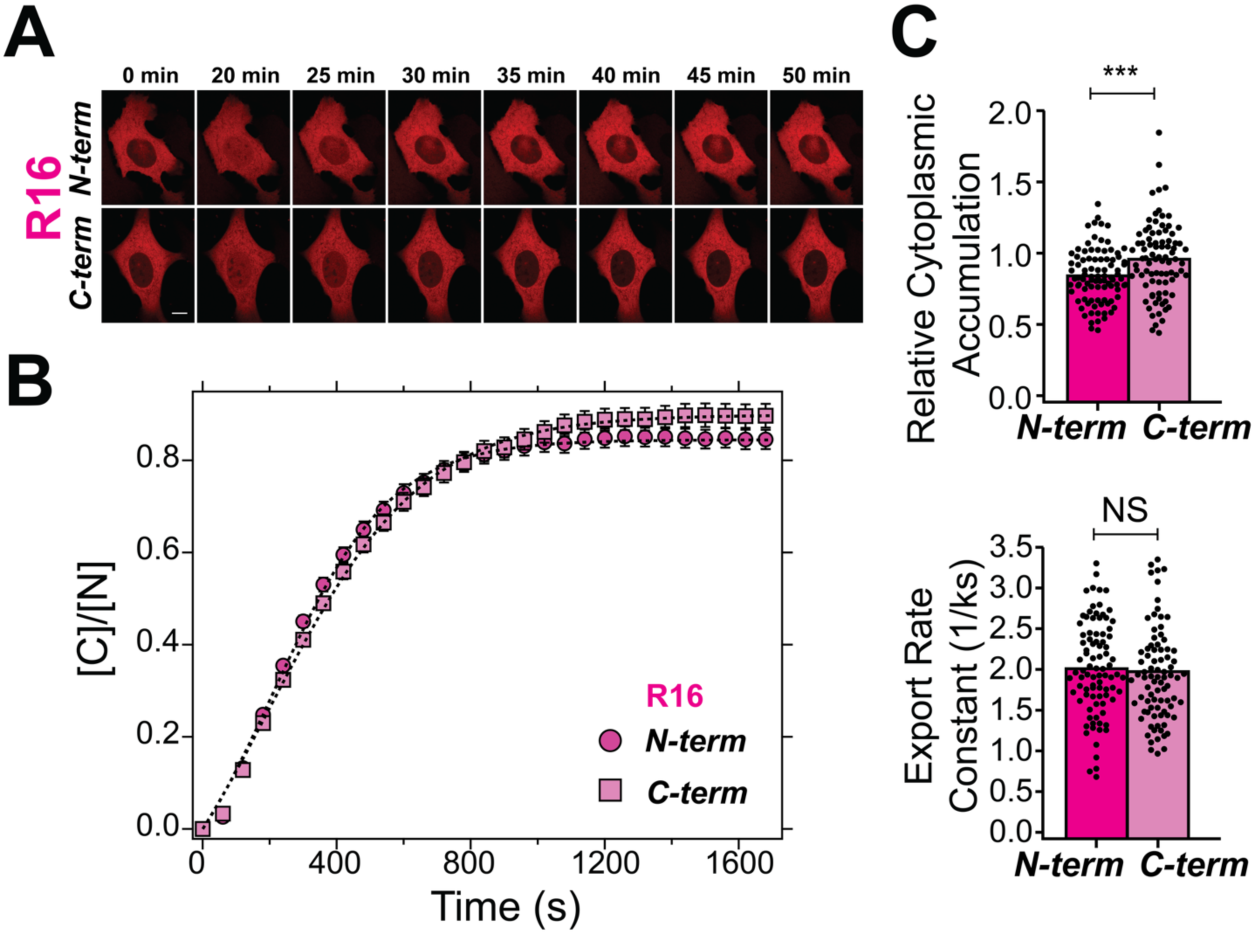

Figure S18. Nuclear export kinetics of R16 when translocating from the N- or C-terminus. (A) Representative confocal images of U2OS cells during the import and recovery phase, scale bar 10 µm. (**B**) Average time-courses of the cytoplasmic-to-nuclear localisation of the "Export forward" and "Export reverse" R16 (**C**) Relative cytoplasmic accumulation (upper) and export rate (lower) calculated from fits to the recovery time courses. Bars indicate mean. Accumulation: N-term K_e=0.84±0.02; C-term K_e=0.96±0.03. Export rate: N-term k_E=1.99±0.03 ks^{-1}; C-term k_E=1.97±0.06 ks^{-1}. Significance levels for two-tailed Mann-Whitney non-parametric test. Accumulation, *P*=7.0x10^{-4}; Export rate, *P*=0.42. *n*=90 (N-term); *n*=88 (C-term) from *N>3* independent experiments.

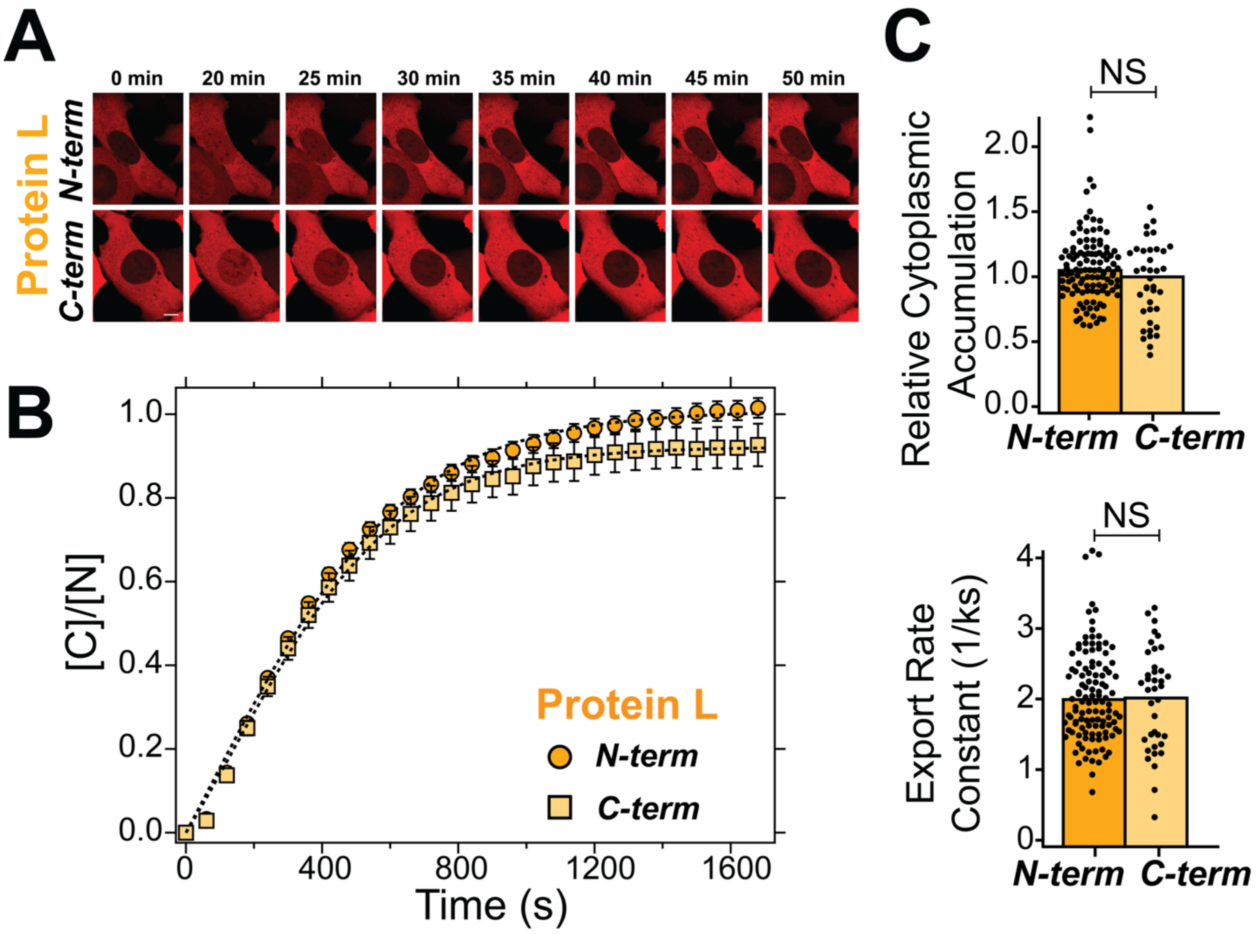

Figure S19. Nuclear export kinetics of Protein L when translocating from the N- or C-terminus. (A) Representative confocal images of U2OS cells during the import and recovery phase, scale bar 10 µm. (**B**) Average time-courses of the cytoplasmic-to-nuclear localisation of the "Export forward" and "Export reverse" Protein L (**C**) Relative cytoplasmic accumulation (top) and export rate (bottom) calculated from fits to the recovery time courses. Bars indicate mean. Accumulation: N-term K_e=1.08±0.03; C-term K_e=0.97±0.05. Export rate: N-term k_E=2.05±0.06 ks^{-1}; C-term k_E=2.01±0.11 ks^{-1}. Significance levels for two-tailed Mann-Whitney non-parametric test. Accumulation, *P*=0.14; Export rate, *P*=0.96. *n*=115 (N-term); *n*=64 (C-term) from *N>3* independent experiments.

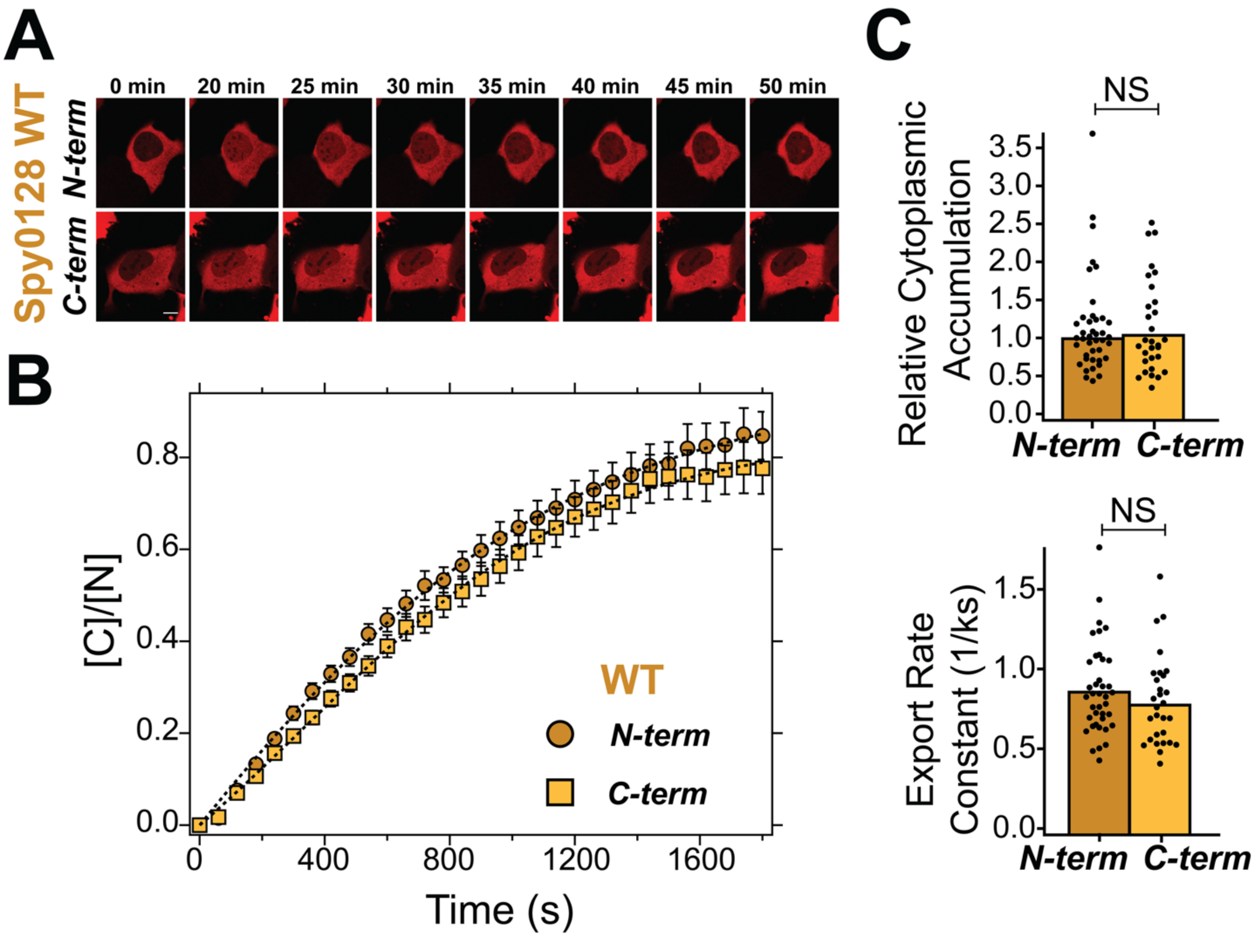

Figure S20. Nuclear export kinetics of Spy0128 WT when translocating from the N- or C-terminus. (**A**) Representative confocal images of U2OS cells during the import and recovery phase, scale bar 10 µm. (**B**) Average time-courses of the cytoplasmic-to-nuclear localisation of the "Export forward" and "Export reverse" Spy0128 WT (**C**) Relative cytoplasmic accumulation (upper) and export rate (lower) calculated from fits to the recovery time courses. Bars indicate mean. Accumulation: N-term K_e=1.14±0.10; C-term K_e=1.13±0.11. Export rate: N-term k_E=0.87±0.04 ks^{-1}; C-term k_E=0.81±0.05 ks^{-1}. Significance levels for two-tailed Mann-Whitney non-parametric test. Accumulation, *P*=0.71; Export rate, *P*=0.33. *n*=41 (N-term); *n*=31 (C-term) from *N>3* independent experiments.

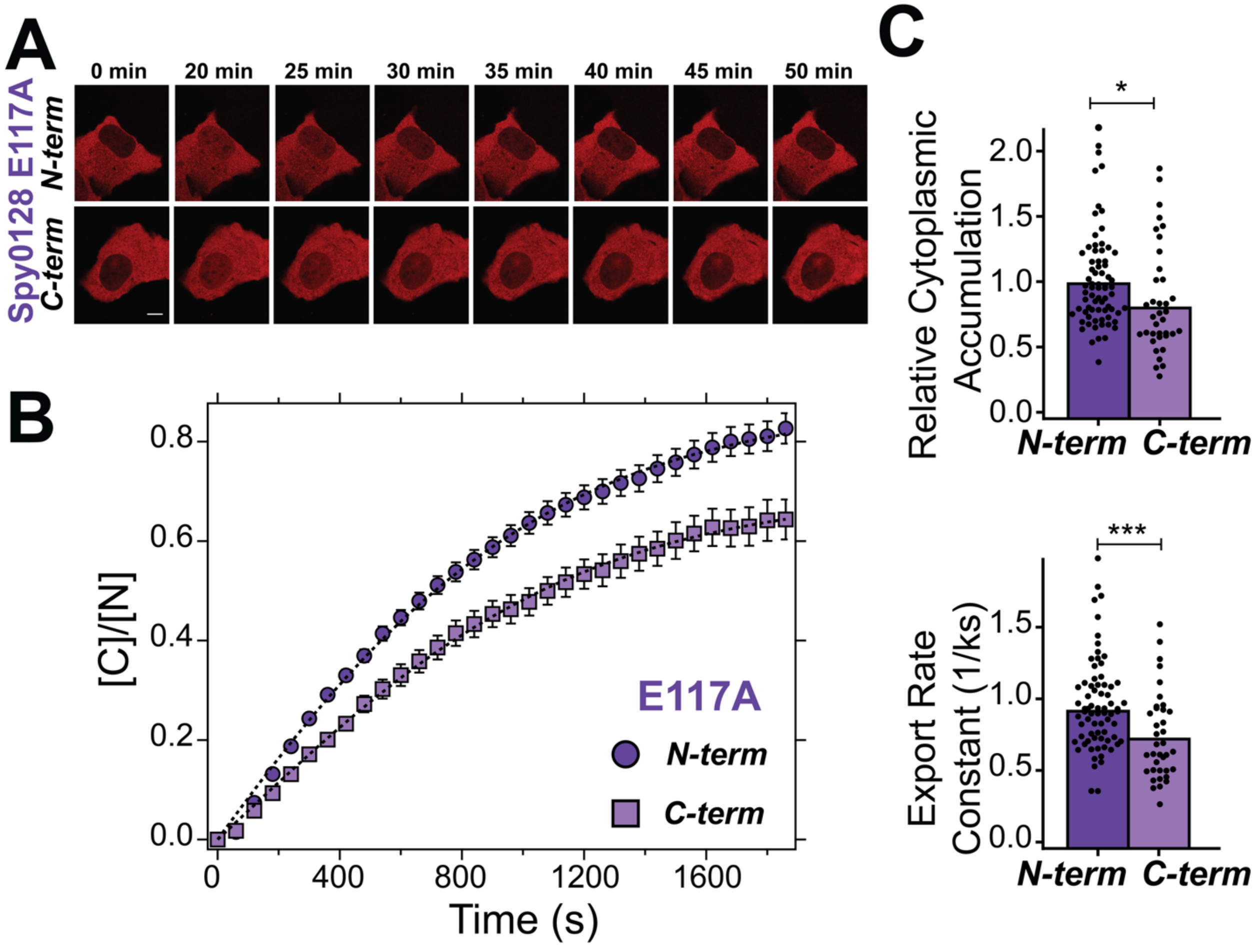

Figure S21. Nuclear export kinetics of Spy0128 E117A when translocating from the N- or C-terminus. (A) Representative confocal images of U2OS cells during the import and recovery phase, scale bar 10 µm. (**B**) Average time-courses of the cytoplasmic-to-nuclear localisation of the "Export forward" and "Export reverse" Spy0128 E117A (**C**) Relative cytoplasmic accumulation (upper) and export rate (lower) calculated from fits to the recovery time courses. Bars indicate mean. Accumulation: N-term K_e=0.99±0.04; C-term K_e=0.88±0.07. Export rate: N-term k_E=0.96±0.04 ks^{-1}; C-term k_E=0.73±0.05 ks^{-1}. Significance levels for two-tailed Mann-Whitney non-parametric test. Accumulation, P=4.1x10^{-2}; Export rate, P=1.5x10^{-4}. n=74 (N-term); n=41 (C-term) from $N>3$ independent experiments.

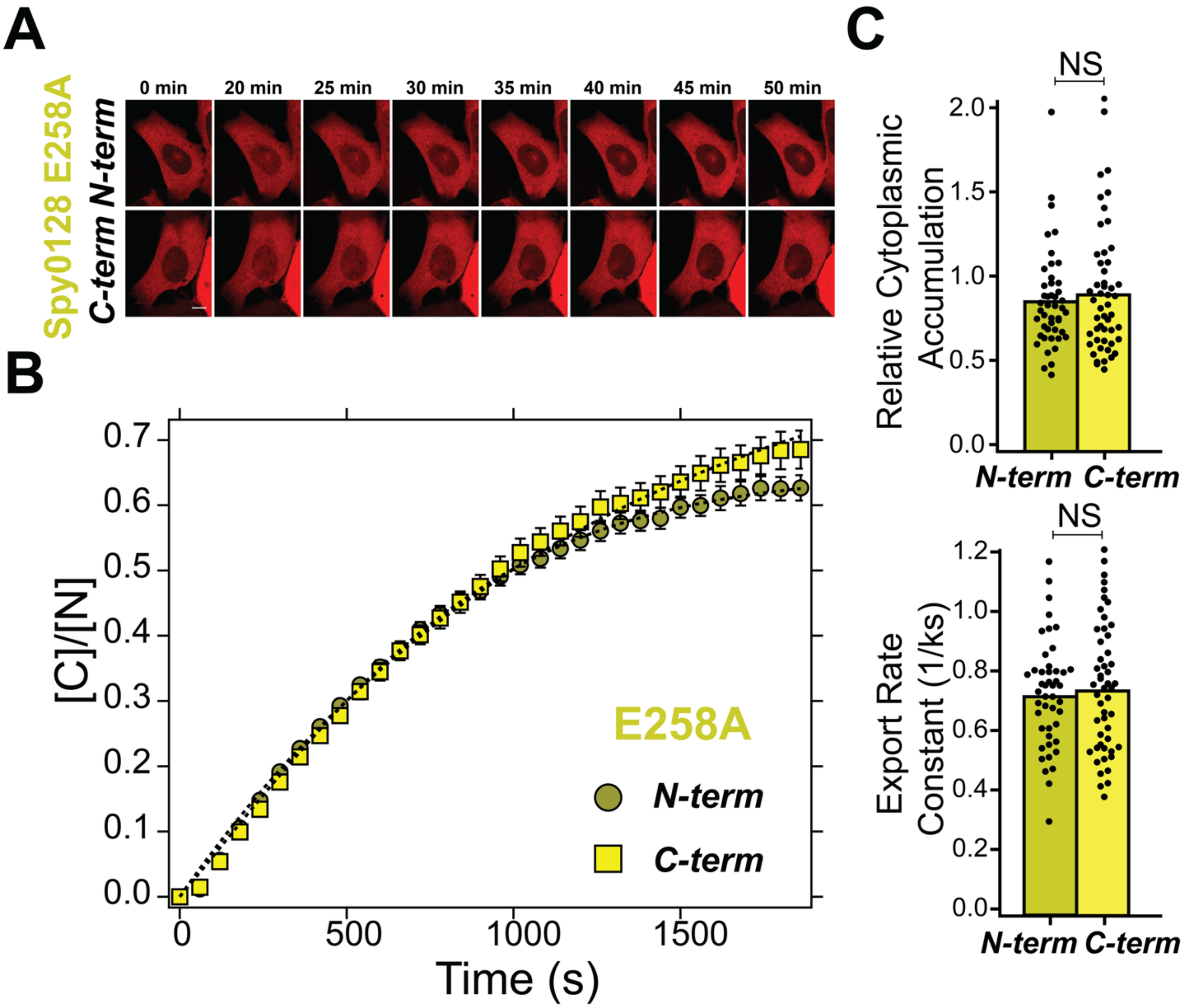

Figure S22. Nuclear export kinetics of Spy0128 E258A when translocating from the N- or C-terminus. (**A**) Representative confocal images of U2OS cells during the import and recovery phase, scale bar 10 µm. (**B**) Average time-courses of the cytoplasmic-to-nuclear localisation of the "Export forward" and "Export reverse" Spy0128 E258A (**C**) Relative cytoplasmic accumulation (upper) and export rate (lower) calculated from fits to the recovery time courses. Bars indicate mean. Accumulation: N-term K_e=0.85±0.04; C-term K_e=0.91±0.05. Export rate: N-term k_E=0.73±0.03 ks^{-1}; C-term k_E=0.75±0.03. ks^{-1}. Significance levels for two-tailed Mann-Whitney non-parametric test. Accumulation, P=0.77; Export rate, P=0.81. n=52 (N-term); n=66 (C-term) from $N>3$ independent experiments.

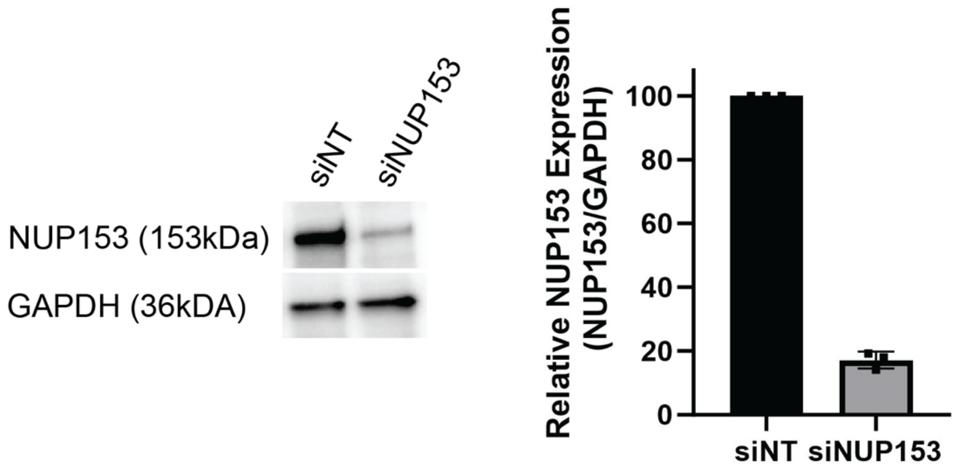

Figure S23. Expression of Nup153 in U2OS in silencing experiments. (Left) representative immunoblotting analysis and (right) relative expression of the corresponding protein normalised by GAPDH. siNT: non-targeting siRNA. siNUP153: Nup153 targeting siRNA. Data points represent independent experiments. Error bars are SD.

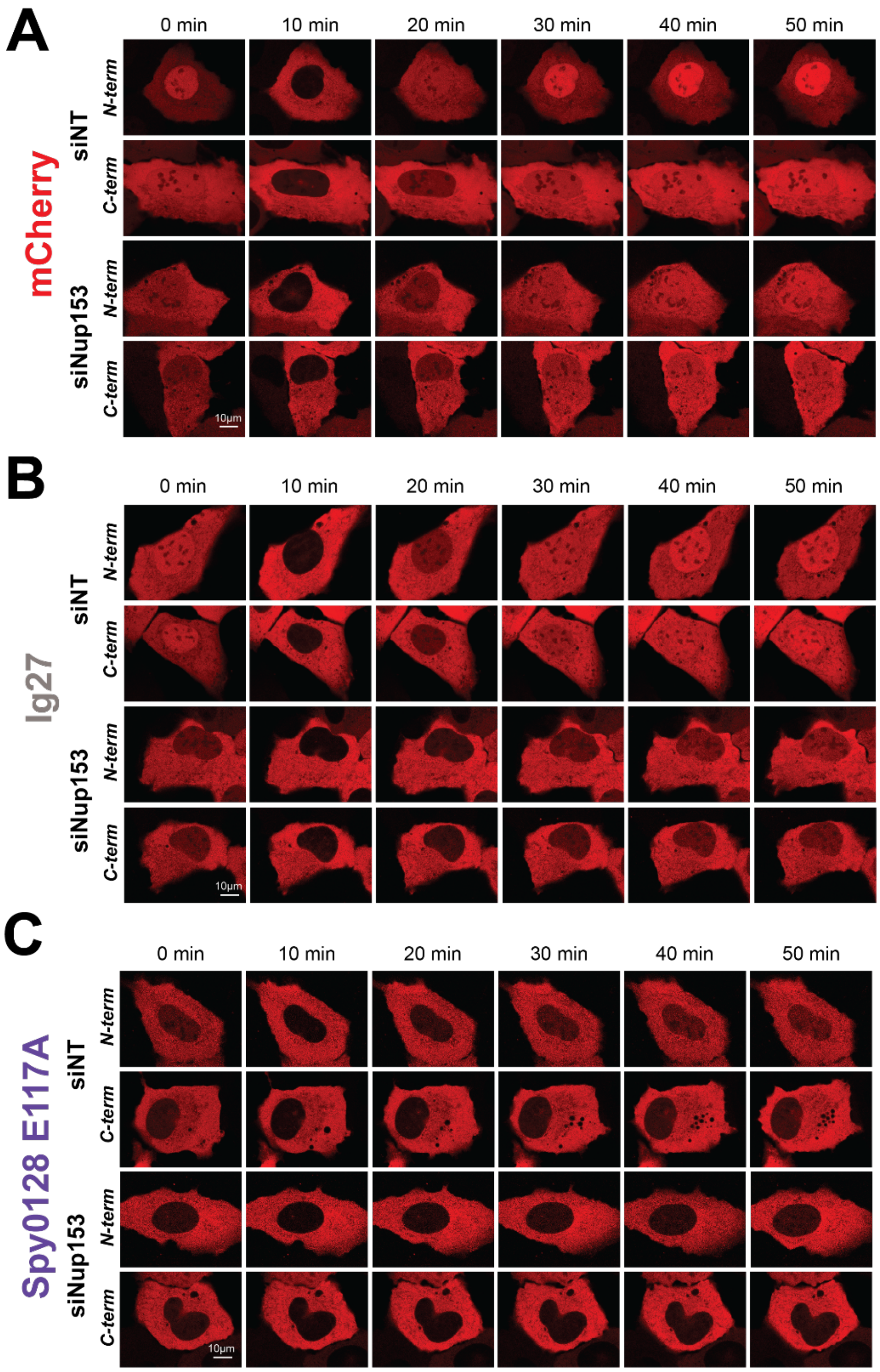

Figure S24. Confocal images of representative U2OS cells overexpressing the (A) "Import forward" (upper) and "Import reverse" (lower) mCherry, (B) Ig27, and (C) Spy0128 E117A optogenetic constructs for the siNT and siNUP153 groups at different time frames.

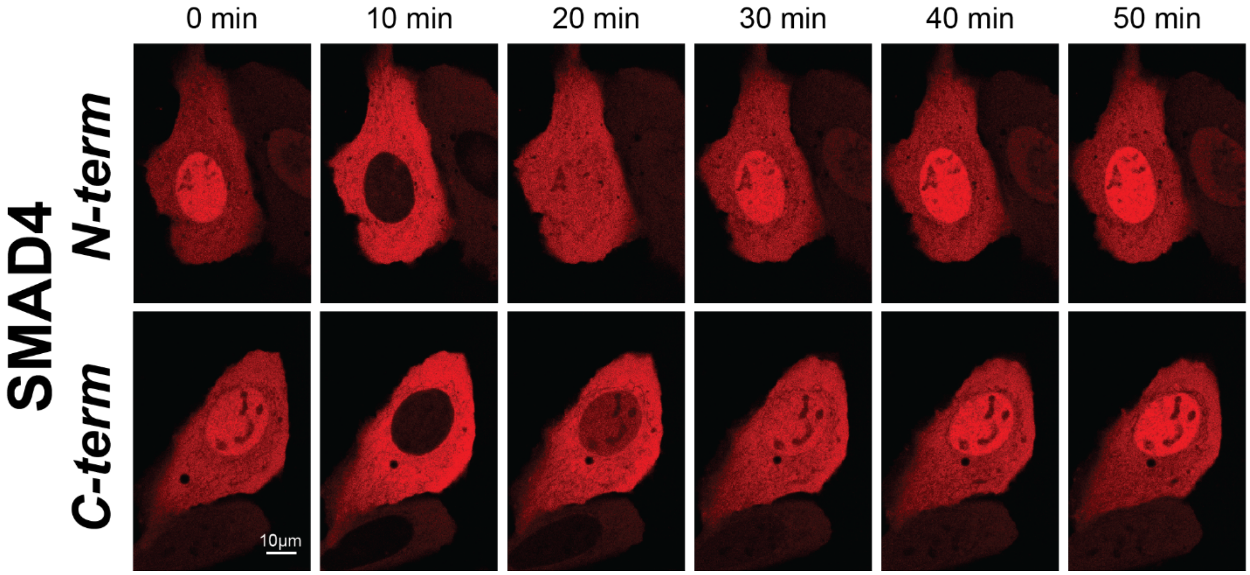

Figure S25. Confocal images of representative U2OS cells overexpressing "Import forward" (upper) or "Import reverse" constructs with SMAD4 as the mechanical sensor. The native NLS and NES of the transcription factor have been inactivated through the use of point mutations. The cMyc NLS is used at either the N- or the C-terminus to provide directional import of the protein, and the LOV2-NES is used to provide light-inducible protein export.

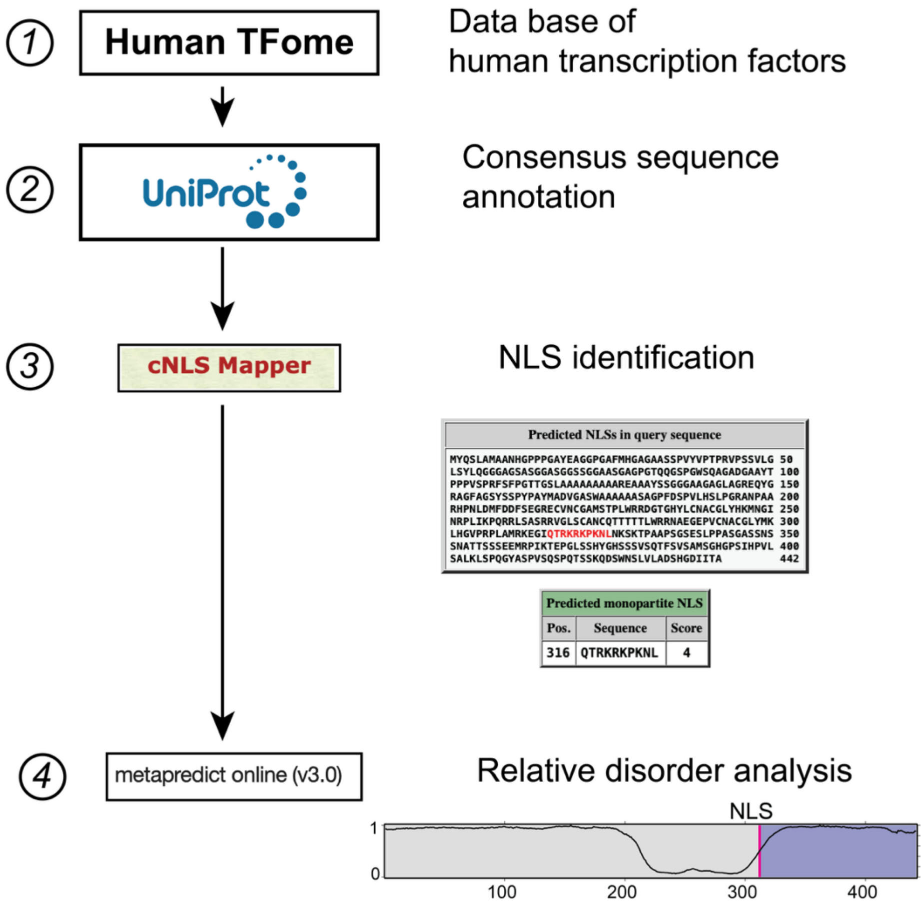

Figure S26. Pipeline for the analysis of the disorder score for the human transcription factors. *1)* The Human TFome is used as the data base for all human transcription factors; *2*) the sequence for each transcription factor is obtained from UniProt; *3)* cNLS mapper is used for identifying NLS sequences on each transcription factor; *4*) the sequence is analysed using Metapredict to extract the relative disorder score across the transcription factor sequence.

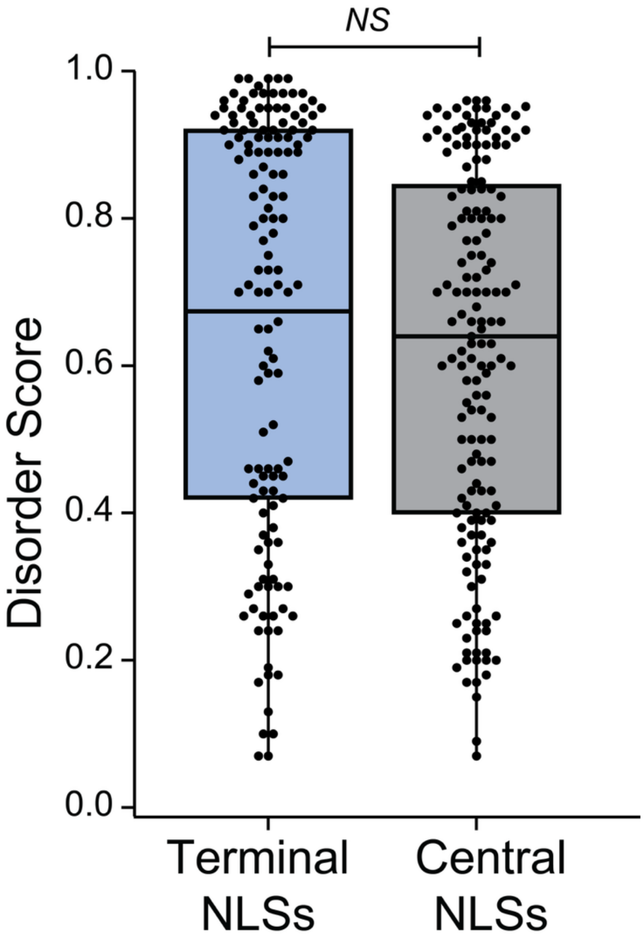

Figure S27: The increased disorder observed in the protein region around the NLS in human TF is not determined by its position within the sequence. Disorder score from the NLS position to the closest terminus for those NLSs located close to a terminus (20% within the sequence, left) and those situated at a central position (20%-50% within the sequence, right). No statistical difference is observed (P=0.09), suggesting that the increased disorder in the region adjacent to the NLS is a feature of NLS-containing sequences, and not of the terminal regions.

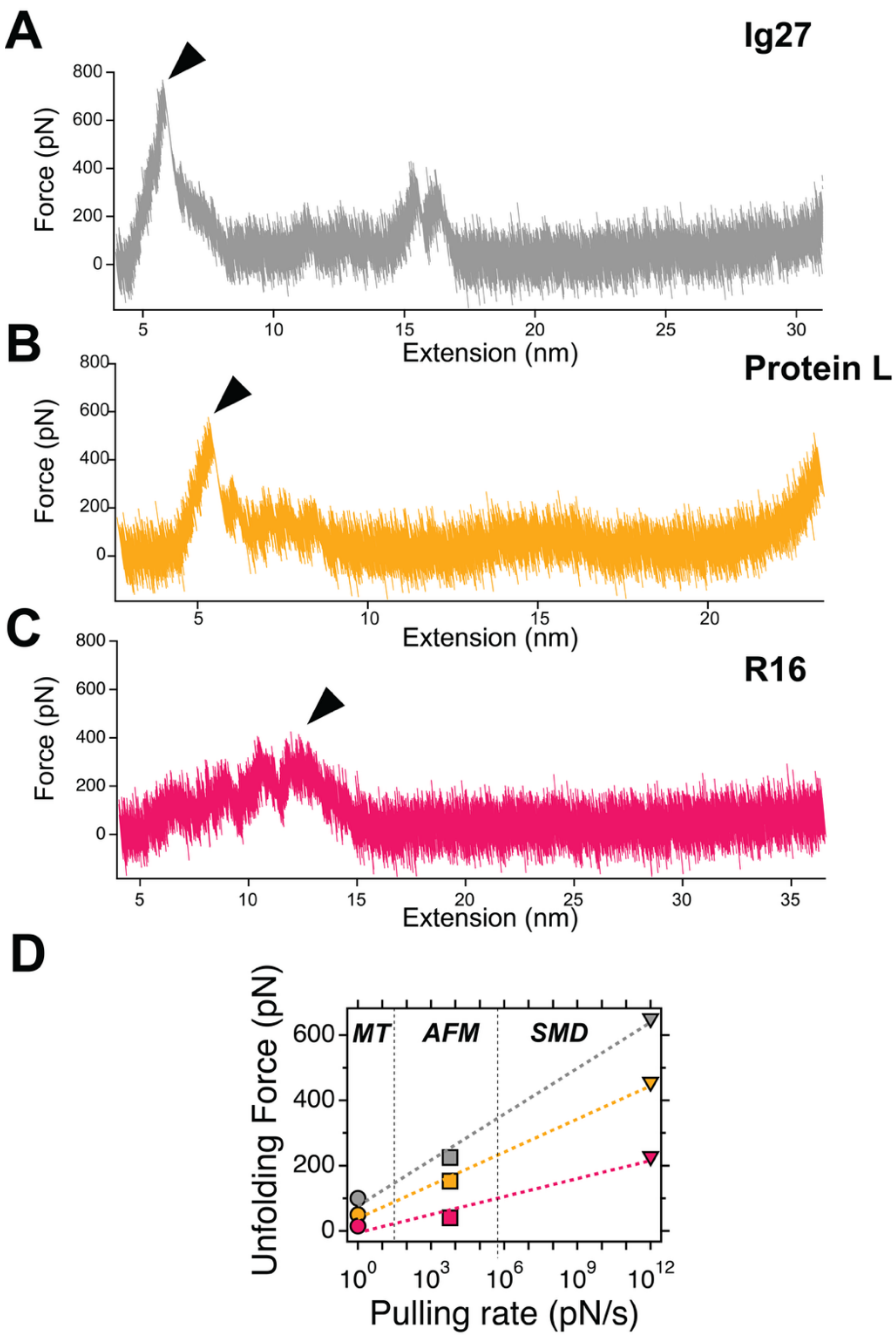

Figure S28. The unfolding force of proteins increases with the pulling rate as measured across different techniques. (A-C) Exemplar force extension trajectories for Ig27 (**A**), Protein L (**B**), and R16 (**C**) from SMD simulations at a pulling velocity of 0.001 nm/ps using a spring with a stiffness of 1,000 pN/nm (pulling rate of 10^{12} pN/s). (**D**) Unfolding rate vs. pulling rate for Ig27 (grey), Protein L (orange), and R16 (magenta) measured in single-molecule magnetic tweezers (pulling rate of 1 pN/s, circles), single-molecule AFM (pulling rate of $6x10^3$ pN/s, squares), and SMD simulations (pulling rate of 10^{12} pN/s, triangles). Unfolding forces increase logarithmically with the pulling rate as predicted by force spectroscopy theories like the Bell model. Each experimental or computational technique operates on a different pulling rate regime, enabling the characterisation of the full nanomechanical spectrum. SMD and MT data was obtained in-house, while AFM data is taken from Li et al.[1] (Ig27), Brockwell et al.[2] (Protein L), and Randles et al.[3] (R16).

Protein	Construct	K_d (nM)	N (sites)	ΔH (kcal/mol)	ΔG (kcal/mol)	$-T\Delta S$ (kcal/mol)
**I27**	**N-term**	535 ± 129	0.87 ± 0.03	-9.98 ± 0.45	-8.42 ± 0.14	1.54 ± 0.57
**I27**	**C-term**	523 ± 116	0.87 ± 0.01	-9.50 ± 0.23	-8.44 ± 0.13	1.06 ± 0.35

Table S1. Thermodynamic parameters from ITC data analysis. Ig27 with either an N- or C-term NLS is injected at 17 μM into 170 μM of importin α3. Mean values obtained from duplicates are reported with standard deviations.

References

1. Li, H., Oberhauser, A.F., Fowler, S.B., Clarke, J. & Fernandez, J.M. Atomic force microscopy reveals the mechanical design of a modular protein. *Proc Natl Acad Sci U S A* **97**, 6527-31 (2000).
2. Brockwell, D.J. et al. Mechanically unfolding the small, topologically simple protein L. *Biophys J* **89**, 506-19 (2005).
3. Randles, L.G., Rounsevell, R.W. & Clarke, J. Spectrin domains lose cooperativity in forced unfolding. *Biophys J* **92**, 571-7 (2007).
